# Chrono-tailored drug delivery systems: recent advances and future directions

**DOI:** 10.1007/s13346-024-01539-4

**Published:** 2024-02-28

**Authors:** Christine T. Butler, Aoife M. Rodgers, Annie M. Curtis, Ryan F. Donnelly

**Affiliations:** 1 Curtis Clock Laboratory, School of Pharmacy and Biomolecular Sciences and Tissue Engineering Research Group (TERG), Royal College of Surgeons in Ireland RCSI, Dublin, Ireland; 2 The Wellcome Wolfson Institute for Experimental Medicine, Queen’s University Belfast, 97 Lisburn Road, Belfast, BT9 7B UK; 3 School of Pharmacy, Queen’s University Belfast, Belfast, BT9 7BL UK

**Keywords:** Circadian rhythm, Drug delivery, Chrono-tailored drug delivery systems

## Abstract

Circadian rhythms influence a range of biological processes within the body, with the central clock or suprachiasmatic nucleus (SCN) in the brain synchronising peripheral clocks around the body. These clocks are regulated by external cues, the most influential being the light/dark cycle, in order to synchronise with the external day. Chrono-tailored or circadian drug delivery systems (DDS) aim to optimise drug delivery by releasing drugs at specific times of day to align with circadian rhythms within the body. Although this approach is still relatively new, it has the potential to enhance drug efficacy, minimise side effects, and improve patient compliance. Chrono-tailored DDS have been explored and implemented in various conditions, including asthma, hypertension, and cancer. This review aims to introduce the biology of circadian rhythms and provide an overview of the current research on chrono-tailored DDS, with a particular focus on immunological applications and vaccination. Finally, we draw on some of the key challenges which need to be overcome for chrono-tailored DDS before they can be translated to more widespread use in clinical practice.

**Graphical Abstract:**

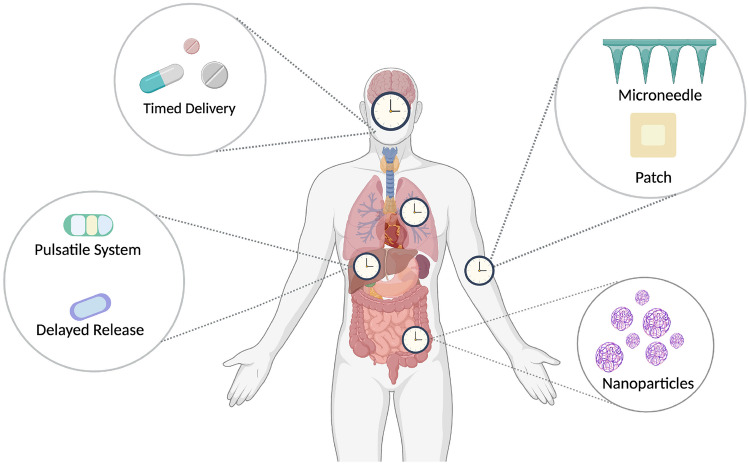

## Introduction

The circadian clock is an internal body or cellular clock with a rhythmic period of 24 h. The central pacemaker, the suprachiasmatic nucleus (SCN), is the dominant oscillator receiving light/dark signals from the eye, and it controls peripheral clocks throughout the body via various signals (Table 1). These clocks or oscillators exist within cells and tissues and are all driven by the same molecular mechanisms as that of the SCN. Almost half the protein-coding portion of the human genome is expressed with 24-h rhythmicity indicating how tightly linked circadian rhythms are with all metabolic, biochemical, and physiological processes throughout the body [1]. The circadian clock tightly regulates various cellular processes including cellular metabolism and the immune response and has a significant influence on the response to various drugs and therapeutics.

**Table 1:** List of circadian terms and definitions

**Circadian terms**	**Definition**
Circadian rhythms	Biological rhythms which follow a 24-hr cycle and exist in the absence of zeitgebers such as light
Zeitgeber / entrainer	Environmental signals which can synchronise biological clocks to their external environment, e.g. light and food
Oscillator	Circadian oscillators are biochemical feedback loops with a period of 24 hrs that can be synchronised with zeitgebers
Diurnal rhythm	Daily rhythms which are synchronised with day/night cycles

**Fig. 1:**
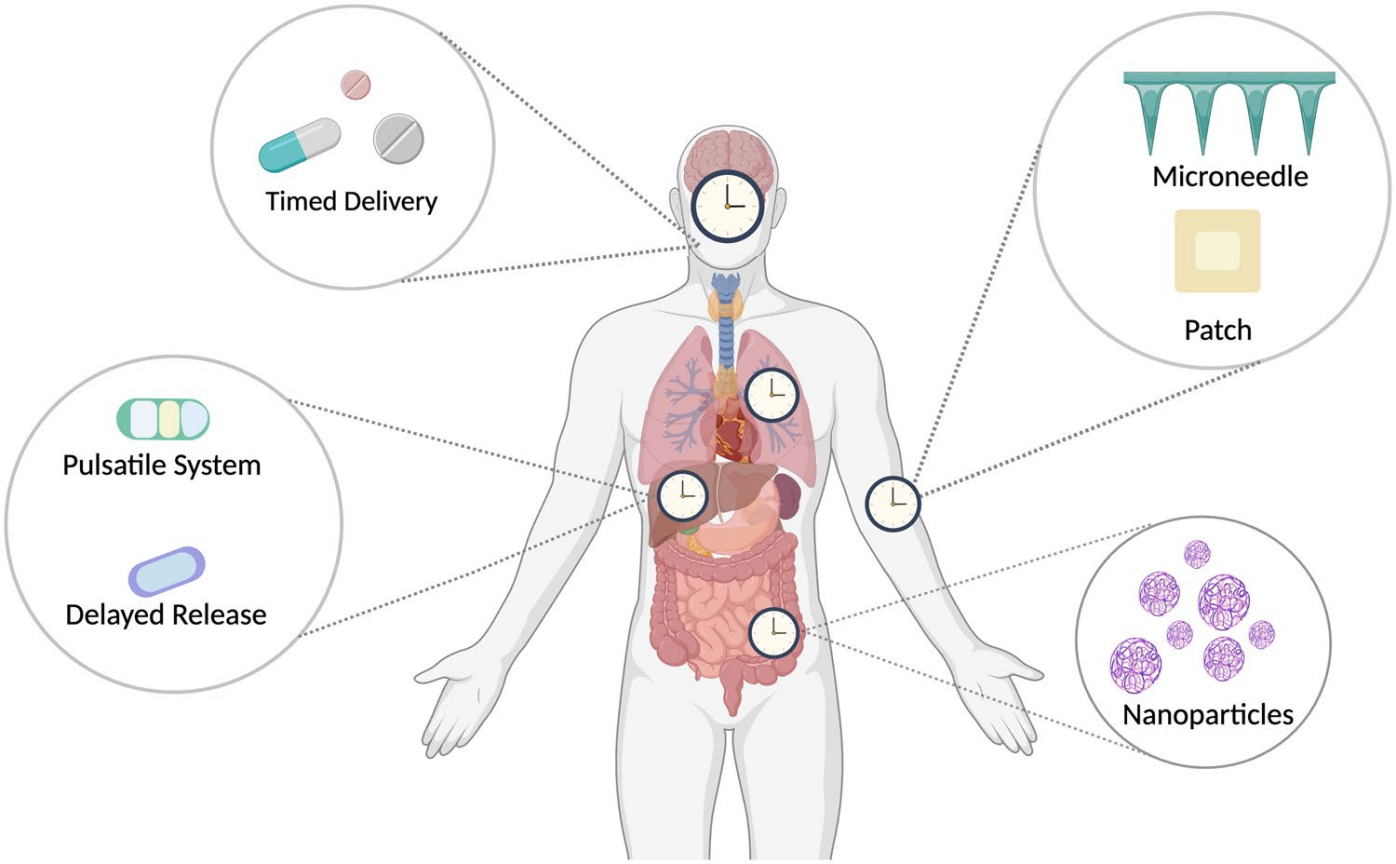
Various methods of chrono-tailored drug delivery systems. Depending on the intended target or organ, various drug delivery systems can be used. These drug delivery systems may also be further modified for time-of-day administration

The clock has wide-ranging control on drug targets and drug metabolising processes, thus optimising drug delivery based on the body’s circadian rhythm has become a point of interest in recent years. Various drug delivery systems currently exist which can be modified for circadian delivery as seen in Fig. 1. These include pulsatile systems, timed and delayed delivery, and transdermal delivery. New technologies such as nanoparticles and microneedles, which are currently under development, similarly show promise to be used in a chronotherapeutic way.

Herein, we first introduce the field of circadian biology and the studies that justify the development of chrono-tailored drug delivery systems (DDS). Following this, we summarise key developments and prospects of chrono-tailored DDS. Finally, we discuss the potential of chrono-tailored vaccination approaches. Overall, we aim to give an overview of the field of drug delivery systems concerning chronobiology and to discuss the prospects and associated challenges within the field.

## Overview of circadian biology

**Fig. 2:**
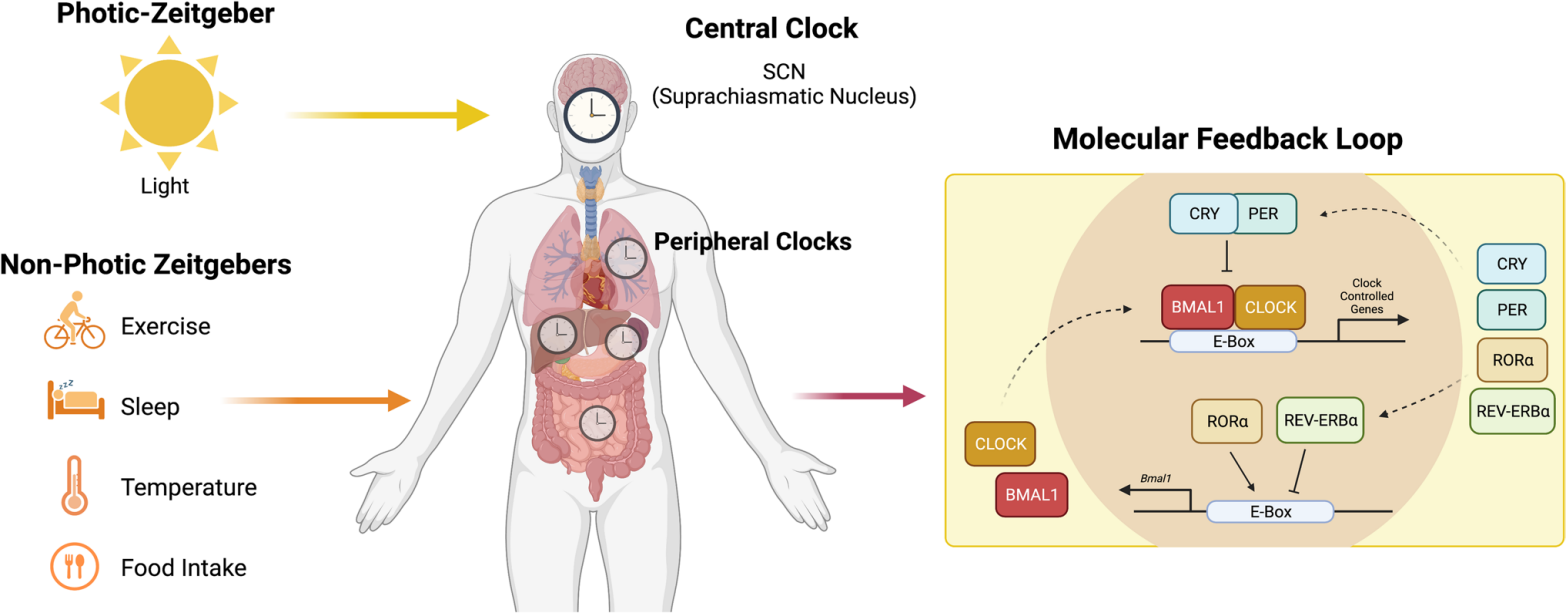
Photic and non-photic zeitgebers influence central and peripheral clocks. The central pacemaker, the SCN, controls peripheral clocks throughout the body. Photic and non-photic zeitgebers act on our central and peripheral clocks. The molecular feedback loop drives circadian rhythm within the cell. BMAL1 and CLOCK bind to E-boxes to induce transcription of clock-controlled genes such as PER and CRY. PER and CRY heterodimerise and move into the nucleus to inhibit their transcription. BMAL1 and CLOCK also regulate nuclear receptors RORα and REV-ERBα, which rhythmically activate or repress the transcription of BMAL1. Created with Biorender.com

Within almost every cell of the body, there exists a 24-h oscillator or circadian clock in which to align one’s physiology and function with the external 24-h world. A central clock exists in the SCN which resides in the hypothalamus of the brain, and receives light/dark signals through photic information conveyed from the retina and transmits them throughout the body [2]. Peripheral clocks exist throughout the body, and the central clock is responsible for coordinating the timing of peripheral clocks through a range of signals either directly or indirectly. These include the autonomic nervous system, hormones, temperature, sleep, and eating habits. These peripheral clocks which include a liver clock, and skin clock coordinate circadian rhythms throughout the body. Circadian rhythms are defined as rhythms with a 24-h period and can exist in the absence of external signals such as light. External light is the most dominant synchroniser of our internal clocks, but clocks can also be synchronised with food, exercise, and temperature [3, 4]. Photic and non-photic zeitgebers act on our central and peripheral clocks as shown in Fig. 2. These synchronisers are often referred to as entrainers or zeitgebers, as they can entrain the internal rhythm to match the external environment. We refer the reader to the following comprehensive reviews on circadian biology and its circuitry [5–7].

## Cogs and wheels of circadian rhythms

Within each cell, the circadian clock and its associated rhythms are driven by transcriptional translational feedback loops. The core of the transcriptional feedback loop consists of two main transcription factors, brain and muscle ARNT-Like 1 (BMAL1) and circadian locomotor output cycles kaput (CLOCK) [8, 9]. BMAL1 and CLOCK heterodimerise within the cytoplasm, translocate into the cell nucleus, and bind to E-box promoters on DNA to regulate mRNA expression of the repressors *Per* and *Cry*, along with a range of clock-controlled genes (CCG) [8, 10–13]. Once translated into protein, PER and CRY then form a complex and translocate back into the nucleus, inhibiting the BMAL1/CLOCK complex [14]. Levels of the PER/CRY complex decrease, causing BMAL1/CLOCK complex binding and activity to increase, which leads to the expression of the repressors once again, and a new cycle begins (Fig. 2). The entire process lasts around 24 h. Other loops within the system include the nuclear hormone receptors Rev-erb (α and β) and Ror (α, β, and γ) which act on ROR/REV-ERB-response element (RORE) rhythmically to repress or activate genes including *Bmal1* [15, 16] (Fig. 2). The circadian clock depends on these core transcriptional/translational loops dictated by the rhythmic expression and degradation of the transcription factors BMAL1 and CLOCK. BMAL1 is of particular focus as the ablation of BMAL1 eliminates the rhythmic expression of core clock genes and causes premature ageing in mice [17, 18]. A more comprehensive description of the molecular mechanisms underpinning the generation of components of the molecular oscillator along with output rhythms in clock-controlled genes has been extensively reviewed elsewhere [19–21].

## Circadian influences on drug metabolism and immune responses

Circadian control exists of many genes involved in the pharmacodynamic and pharmacokinetic processes. Ayyar and Sukumaran recently discussed in detail the emerging field of chrono-pharmacology and how the clock can influence the four processes of pharmacokinetics; absorption, metabolism, distribution and elimination (ADME) and toxicity [22]. We will discuss in further detail ADME and toxicity and how circadian control of these processes justifies chrono-tailored DDS. Further, we will discuss how the clock influences the immune system and how the immune system is amenable to chrono-tailored DDS.

### Absorption

Oral administration of drugs is the most widely used route of administration due to its simplicity. However, drugs must contend with the physiological processes involved in the gastrointestinal tract. Physiological processes such as gastrointestinal pH and blood flow along with gastric emptying have been shown to have a circadian fluctuation which all influence drug absorption [23, 24]. The role of the clock has been shown to be interlinked with nutrition, with diurnal variation in various transporters that are involved with lipid, carbohydrate, and protein uptake [25–27]. CLOCK, one of the main components of the core circadian clock, has also been shown to regulate macronutrient absorption [28]. Diurnal variation in nutrient uptake is therefore likely to influence the uptake and absorption of drugs [29]. Lipophilic drugs are shown to be affected by time of day, with faster absorption in the active versus the rest phase. Valproic acid, which is a lipophilic drug used to treat epilepsy, displays time-of-day differences in drug absorption and concentration. A previous study examined the maximum absorption times of valproic acid at different times of day in mice and found that maximum drug concentration in the blood ranged from 386 ± 30.86 mg to 824 ± 39.85 mg during the rest and active phase, respectively [30]. Time of day differences in gastric emptying may also account for variations seen in drug absorption between morning and evening. A previous study with 16 healthy male volunteers showed that gastric emptying in the evening was significantly longer than in the morning [31]. The gastrointestinal tract, which is the most widely used and simplistic method of drug delivery, is influenced by the clock in many aspects. The absorption process should also be considered for the drug delivery to the skin. Factors such as skin pH and perfusion change throughout the day which can influence the absorption of drugs [32, 33]. Diurnal variation has been found in a water transporter in the skin in wild-type mice and HaCaT cells, which suggests the oscillations in epithelial barrier function could play a role in transdermal drug absorption [34]. However, there are no studies directly comparing drug absorption in the skin and the clock.

### Distribution

Drug distribution is an important factor in pharmacokinetics; however, modelling drug distribution is difficult making it the lesser studied of the four pharmacokinetic phases [35]. Distribution concerning the clock is also an understudied area; however, there is indirect evidence of clock involvement. The clock is shown to be involved in both blood flow and plasma protein availability two factors which distribution relies on [36]. In terms of blood flow, cardiac output shows diurnal variation with increased cardiac output and blood flow in the morning and decreasing blood flow and hepatic blood flow in the evening [37]. Distribution also relies on plasma protein availability. Drugs bound to plasma proteins are inactive, while unbound drug exists in the active form. Therefore, the affinity of the drug to bind to plasma proteins will affect its distribution and activity. Circadian variation in the concentration of various plasma proteins has previously been shown [38, 39]. This circadian variation in plasma proteins has also been shown to correlate with drug distribution when using valproic acid and chemotherapeutic agents [30, 40, 41]. Thus, the clock is associated with changes in blood flow and plasma protein levels, two factors which drug distribution relies on. However, currently, there is no strong evidence of direct circadian variation in drug distribution.

### Metabolism

The liver and the kidneys are the main organs involved in drug metabolism. Metabolism in the liver of mice has been shown to be under the control of the clock, with 335 transcripts cycling in mouse livers [42] and 50% of metabolites found to be cycling [43]. Within the liver, PAR-domain basic leucine zipper transcription factors DBP, TEF, and HLF are found to accumulate in a circadian manner [44]. These transcription factors interact with genes that control the expression of enzymes which regulate metabolism in the liver. Various metabolic pathways including xenobiotic detoxification, glucose, lipid, and carbohydrate metabolism have all been linked to the clock within the liver [45–47]. Research on metabolism in the kidney has suggested that it is under the control of the renal tubular circadian clock [48]. However, further research is needed to understand the effects of the clock on metabolism in the kidney.

### Elimination

Elimination and excretion of therapeutics are mostly carried out by the kidneys. Diurnal variation in excretion was shown to occur in healthy individuals with rhythmic excretion of proteins such as albumin, transferrin, and immunoglobulin [39, 49, 50]. Renal excretion of electrolytes and minerals such as calcium and magnesium have further been shown to be under the influence of the clock [51, 52]. Urinary pH in humans has been shown to vary with the time of day which may account for variations in drug excretion through the day [53]. Although there are variations in kidney function and elimination of metabolites associated with circadian rhythm, the exact mechanism is unknown. Therefore, to understand the precise role of the clock in drug elimination, we need to further unravel the exact mechanisms by which the clock influences kidney and liver.

### Toxicity

Toxicity is a major concern when drugs are administered to patients. Efforts to reduce toxicity may include reducing dosage, but importantly, research suggests altering the time of day of administration may also influence drug toxicity. It has been shown previously that liver cells’ (hepatocyte) circadian rhythm alters the xenobiotic metabolism [54]. Xenobiotic metabolism is responsible for the removal of foreign or toxic substances that are not normally produced by the body. With disruption of the clock in the liver, xenobiotic metabolism is altered leading to greater or lesser detoxification depending on the clock components altered. Further, hepatocytes’ circadian rhythm has also been shown to affect acetaminophen toxicity [55]. In the kidney, tobramycin administration in rats shows varying nephrotoxicity during excretion depending on the time of day of administration [56].

Large databases show that a number of tissue-specific genes are rhythmically expressed. This data provides further rationale for application to circadian medicine. Interestingly, many key cardiovascular genes are rhythmically expressed, which is of particular importance for drugs which may have off-target cardiovascular effects [1]. Bronchodilators are known to have off-target effects, by interacting with genes that are important in cardiovascular function, causing major adverse cardiac effects, and this is a major issue in their use [57]. Therefore, timing the administration of bronchodilators to coincide with the rhythmic downregulation in off-target cardiovascular genes may prevent cardiotoxicity.

**Fig. 3:**
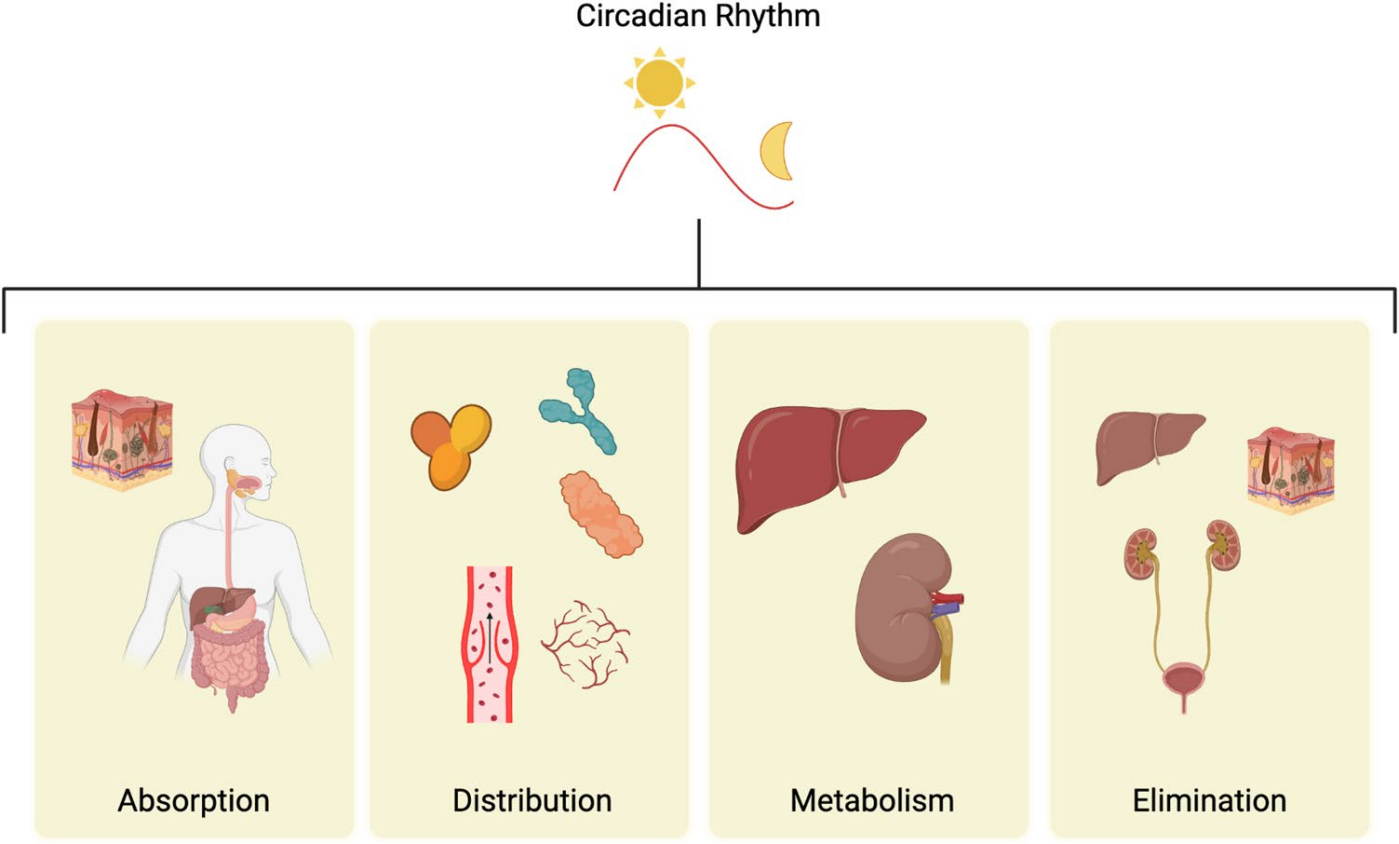
Impact of the clock on absorption, distribution, metabolism, and elimination. The circadian clock impacts pharmacodynamics and pharmacokinetics at various stages through different organs and processes. Absorption occurs primarily through the GI tract and the skin. Distribution is influenced by plasma protein levels, blood flow, and tissue perfusion. Metabolism and elimination are mainly carried out by the liver and kidneys, with some elimination occurring through the skin. Each of the stages is influenced by the clock with 24-h rhythms in numbers or functions. Created with Biorender.com

Thus, the clock appears to influence aspects of drug pharmacodynamics and kinetics in humans, as seen in Fig. 3. However, this area of chrono-pharmacology is still in its infancy. More detailed studies of this topic can be found here [22, 58, 59].

### Circadain immune system

The immune system is one system that is particularly under the influence of the circadian clock and thus may be particularly amenable to a chrono-tailored DDS approach. For example, many immune cells display circadian alterations in number in the body [65]. Members of the innate and adaptive immune system, including dendritic cells (DC) [66, 67], macrophages [68, 69], B cells [70], T Cells [71], and monocytes [72], all display circadian oscillations in clock genes and their outputs. For many years, researchers have been investigating the reasons why immune cells alter their phenotype throughout the time of day. Having immune cells primed for activation during the active phase, when the host is most likely to encounter a pathogen or become injured, versus during the rest phase, when the chance of infection or injury is lower, may be the reason. Whatever the underlying cause for why this mechanism evolved, it is well established that challenging an organism with lethal amounts of lipopolysaccharide (LPS) at varying times of the day leads to different host responses. Indeed, LPS challenge in mice as they approach their active phase is much more lethal than at the start of their rest phase [73]. Furthermore, evidence of rhythmic trafficking of immune cells between the skin, blood, and lymphatics has been elucidated [65, 67]. Circadian expression of chemokine receptors and adhesion molecules aid the circadian movement of immune cells between different organs. Similarly, the metabolism of immune cells varies depending on the time of day [66]. Recent reviews have covered the circadian immune system; in more detail [65, 74], we will discuss further the immune system in relation to chrono-tailored DDS.

## Overview of drug delivery systems amenable to circadian rhythm

Therefore, understanding the role of the circadian clock in pharmacodynamic and pharmacokinetic phases is required to develop appropriate chrono-tailored drug delivery systems (DDS). In line with this, selecting the correct drug delivery system to align with the clock and chrono-pharmacology is also key. Depending on the drug and its intended use, the target tissue or organ, dosage, and release profile, different delivery systems can be exploited for maximum benefit. It is important to consider the method of delivery, to effectively align drug delivery with the appropriate clock physiology. As previously discussed, absorption is influenced by the clock and is largely dependent on the site of administration. The most common method of drug delivery is in capsule or tablet format. The time of administration and the structure of the tablets can be altered to align more efficiently with the changes in absorption and metabolism of the GI tract. However, other methods of drug delivery may be more easily adapted for chrono-tailored delivery. Transdermal delivery is an exciting avenue for chrono-tailored DDS. Recent advances in microneedle technology have the potential for self-application at the most appropriate time of day. Nanocarriers such as liposomes, niosomes, and polymeric nanoparticles also have the potential for chrono-tailored DDS, as they can be targeted more efficiently to specific sites. Increased site specificity along with an understanding of the chrono-pharmacology at that site offers an advantage of increased drug delivery and the potential of less toxicity. It is important to consider the sites of action, the method of delivery, and the interplay between the drug delivery system and the clock. We will discuss further the various drug delivery systems and their potential benefits as a chrono-tailored DDS.

## Timed and chrono-tailored drug delivery systems (DDS) and their application in disease

**Fig. 4:**
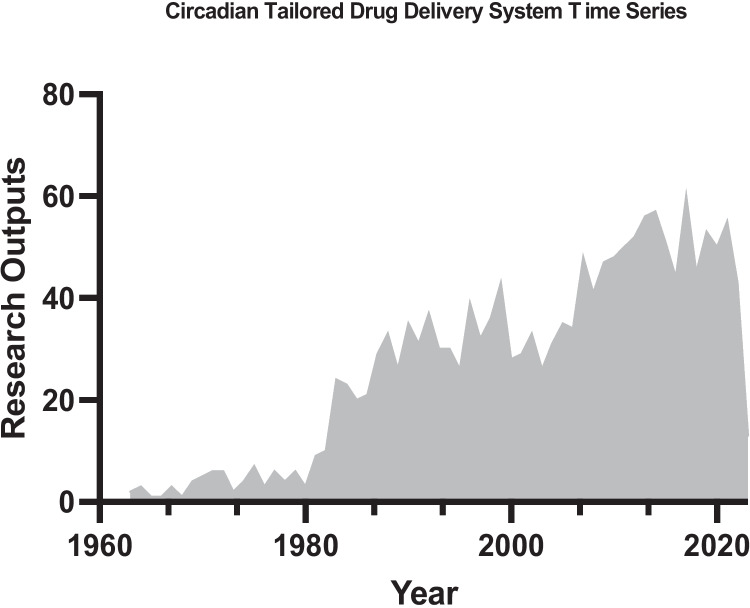
Circadian-tailored drug delivery system research trend. Time series chart illustrating current research trends of chrono-tailored drug delivery systems reported in the literature, 1960–2023, (*n* = 1510). Derived from www.ncbi.nlm.nih.gov/pubmed/. Search query: “(circadian OR chrono OR Timed) AND Drug Delivery)”

Chrono-tailored DDS draws on the principles of drug delivery, pharmacology, and chronobiology. It is inherently interdisciplinary and requires input from a wide range of scientific fields. Tailoring drug delivery to the clock is a relatively recent idea that has been steadily gaining interest (Fig. 4). Moreover, in recent years, researchers have begun to develop and evaluate circadian-tailored drug delivery systems to improve the therapeutic efficacy of drugs and vaccines and avoid potential side effects. Coordinating drug delivery with the clock to coincide with the increase in the intended receptor or decrease in the potential off-target receptors is beneficial to increase the therapeutic index and decrease toxic side effects. For example, chemotherapeutic agents have been shown to be more effective and produce less toxic side effects when given at certain times of the day; however, the exact mechanisms and understanding of chemotherapeutics and chronobiology require further exploration [60–62]. In the subsequent sections of this review, we discuss some of the key approaches researchers have used to harness the circadian rhythm for optimal time-of-day delivery of drugs and vaccines.

### Approved market products and patents

There are several approved products on the market for chronotherapeutic applications. Modified release Prednisone, as previously mentioned for the treatment of rheumatoid arthritis, is currently on the market under the name Rayos^®^. CODAS^®^ and VERELAN PM^®^ both utilise Verapamil nighttime delayed release for hypertension. PULSINCAP^®^ is also a registered system that utilises the pulsatile system to create lag times in drug release. GEOMATRIX^®^ uses multiple layers to cause delayed release or pulsatile release of drugs. While there are some products currently on the market, there are also many patents filed and granted for chronotherapeutic drug delivery. Below is a list of patents which have been published in the last 10 years, which are drug delivery systems that offer varied release profiles with the aim of being chrono-therapeutics (Table 2). Information on patents was collected using “The Lens” free and open patent and scholarly search portal.

**Table 2:** Table of patents published from 2010 to 2013, detailing drug delivery systems that offer varied release profiles to be used as chrono-therapeutics. Information collected using Lens.org

**Jurisdiction**	**Display key**	**Publication year**	**Title**	**Abstract**
US	US 11672697 B2	2023	Eye-mountable therapeutic devices, and associated systems and methods	Eye-mountable devices to facilitate chronotherapeutic treatment of primary open-angle glaucoma
US	US 11596779 B2	2023	Drug delivery methods and systems	A two-part bioactive agent transdermal delivery system
US	US 11596607 B2	2023	Polymer-based formulation for release of drugs and bioactives at specific GIT sites	Polymer-based formulation for release of drugs and bioactives at gastrointestinal tract-specific sites
US	US 11471424 B2	2022	Biosynchronous transdermal drug delivery	Systems and methods for synchronising the administration of compounds with the human body's natural circadian rhythms
US	US 11147772 B2	2021	Timed, pulsatile release systems	Multiparticulate dosage form for delivering pulsatile timed delivery
US	US 11065205 B2	2021	Immediate/delayed drug delivery	Novel formulations for an immediate, followed by a delayed release of drug
CN	CN 112703035 A	2021	Chronotherapeutic treatment profiling	A method of electromagnetic energy delivery for specific timing of drug delivery
US	US 10874615 B2	2020	Formulation having controlled, delayed release of active ingredient	Novel pharmaceutical formulations which have controlled, delayed release of active ingredient
US	US 10624858 B2	2020	Controlled-release composition using transition coating, and method of preparing same	Transition coating to control and or target the release of active pharmaceutical ingredients
US	US 10561602 B2	2020	Controlled extended-release pregabalin	A controlled extended-release composition
US	US 9561188 B2	2017	Controlled-release delivery device comprising an organosol coat	A controlled-release delivery device for controlled release of an active ingredient
US	US 9504640 B2	2016	Modified release formulations of a bupropion salt	Modified release tablets
US	US 9474719 B2	2016	Pulsatile drug release	Novel formulations for a delayed, followed by a pulsed release of drug
US	US 9283192 B2	2016	Delayed prolonged drug delivery	Novel formulations for a delayed, followed by a prolonged release of drug
US	US 9125803 B2	2015	Gastric release pulse system for drug delivery	Pulse of at least one pharmaceutically active ingredient in stomach, or from a subsequent gastrointestinal site
MY	MY 153027 A	2014	Combined preparation for the treatment of cardiovascular diseases based on chronotherapy theory	Combination preparation for enabling two drugs to be chronotherapeutically released
US	US 8673352 B2	2014	Modified release dosage form	Inventive dosage forms provide modified release of one or more active ingredients
US	US 8394409 B2	2013	Controlled extended drug release technology	A controlled extended drug release technology
US	US 8389008 B2	2013	Delayed-release dosage forms	A delayed-release pharmaceutical formulation
US	US 8372040 B2	2013	Portable drug delivery device including a detachable and replaceable administration or dosing element	A device for transdermal drug delivery and administration of differing dosages at specific times of the day

### Timed delivery

One of the most simplified strategies to effectively target drugs to align with circadian rhythms is through direct administration of treatments according to the clock on the wall. An early study by Hamprecht et al. showed that cholesterol synthesis displayed a circadian rhythm [63]. This, in turn, led to Simvastatin, a short-acting cholesterol-reducing agent, being prescribed for patients to be taken in the evening. This is one of the first cases where circadian biology was translated into medical care to increase drug efficacy, with significant differences found in the effectiveness of lowering cholesterol if taken in the evening versus the morning [64].

With respect to immunological diseases, timed delivery of drugs has been shown to be effective in conditions such as asthma. Asthma shows strong circadian variation with symptoms being worse and more prevalent at night than in the morning [75, 76]. In one study, the administration of steroids to patients with moderate and moderate to severe asthma was randomised into two groups. One group received one dose of inhaled steroids at 3 pm, while the second group received four doses at 7 am, 12 pm, 7 pm, and 10 pm. Both groups received a total of 800 µg of steroids per day. They found no increase in overall toxic systemic effects of the drug with both regimens, but that efficacy was still found to be similar between both groups [77]. This is incredibly beneficial as not only is it more cost-effective to reduce the number of dosages per day, but more importantly, it can lead to higher patient compliance [78]. However, one of the main challenges surrounding the timed delivery of drugs is that it requires patients’ consistency with the administration of drugs at the optimal time of day.

Sancar et al. detail the intricate links of the circadian rhythm coupled with cancer and chemotherapy and conclude that is worth exploring the link between circadian timing of chemo and radiotherapy to try and improve patient outcomes [79].While there are defined links between DNA damage susceptibility and cell cycle changes and the circadian clock, currently, the evidence regarding what time of day is most efficient for chemotherapy is lacking [80–82]. A study looking at B cell lymphoma in female patients found differences in overall survival and progression-free survival between morning and afternoon administration of chemotherapy [83]. Indicating that in this cancer subtype in female patients, there appears to be a benefit to time of day administration, but this cannot be generalised across all cancer subtypes. Some studies have shown that time of day administration of chemotherapy and radiotherapy is more effective for patients in terms of increasing efficacy and decreasing unwanted side effects [60, 84]. However, it is hard to define the exact time of day that chemotherapy should be administered as it appears to depend on several factors including the drugs administered and the cancer subtype. Papers as far back as the early 1990s discuss the implications of chrono-modulation in cancer therapeutics [62, 85]. While some links exist, further studies are warranted for different chemotherapeutic agents and their specific time-of-day effects. Similarly, results may vary based on the patient’s disease status, age, sex, and the different combinations of chemotherapeutic drugs patients are taking. It is a tough endeavour as both circadian interplay with cell cycle and DNA repair needs further exploration, and disruption of the clock within tumours also warrants further studies. There is much research to be done to fully comprehend the complex interplay between cancer, chemotherapy, and the circadian rhythm to precisely define the correct time of day of administration.

One of the most significant barriers to administering not just chemotherapeutic agents but other drugs at the correct time of day in a clinical setting is the scheduling, availability, and coordination with healthcare professionals. Therefore, the main drawbacks of timed delivery of chemotherapeutics are patient compliance and scheduling and, in the healthcare setting, the practicalities of timed delivery.

### Delayed release and pulsatile delivery

**Fig. 5:**
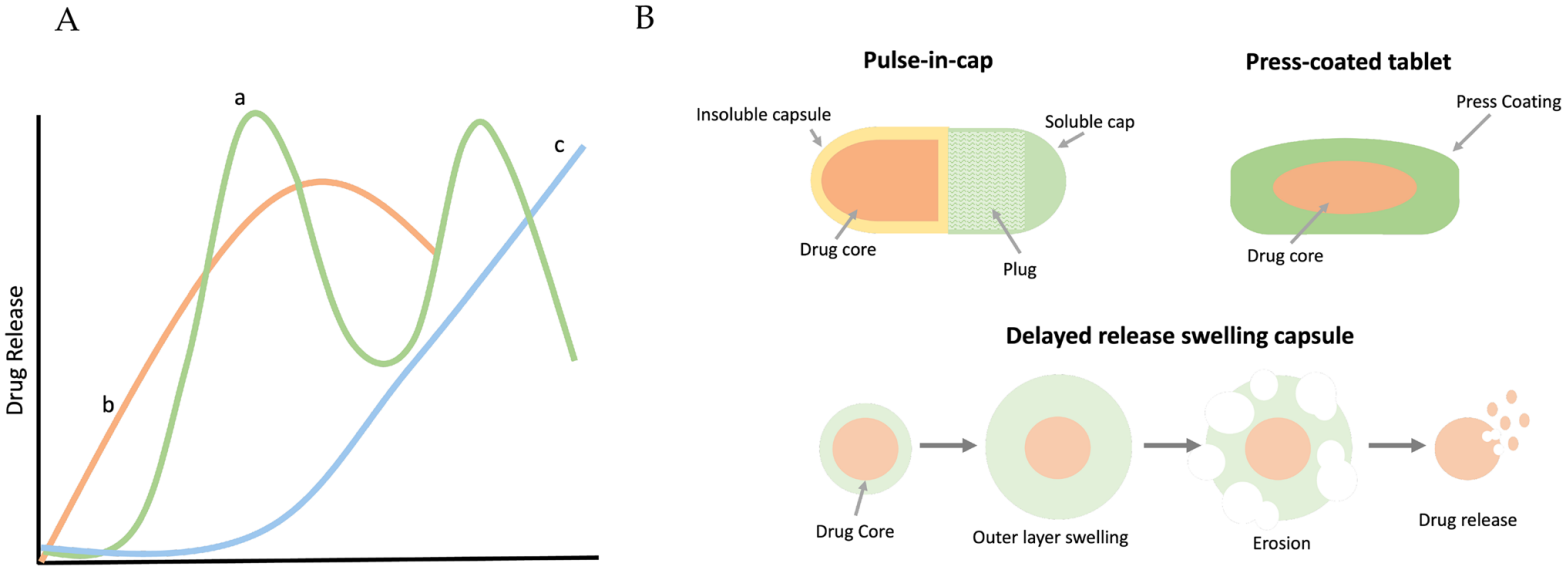
Drug release from pulse-in-cap, press-coated tablets, and delayed-release capsules. Graph depicting drug release and schematic of pulsatile drug release capsules. **A** Drug release over time, (a) pulsatile release, (b) conventional drug release, (c) delayed release. **B** Schematic of pulse-in-cap capsule, press-coated tablet, and mechanism of super swelling delayed-release capsule. Created with Biorender.com

Similar to timing the delivery of drugs, pulsatile and delayed release offer the slow release or subsequent release of drugs at the appropriate time. A delayed or pulsatile release can be achieved through the modification of tablet coatings or patches [86–88]. Different methods of modifying tablet coatings for delayed release are available. While some delayed-release tablets can be repurposed for chrono-tailored drug delivery, other tablet formulations have been specifically developed for chrono-tailored DDS [89, 90]. Coating technology is generally applied to delay the release of tablets. Film coatings are used to thinly cover the tablet with a polymeric film which protects the tablet from degradation within the stomach in the instance of delayed release; coatings are usually used to prevent instant degradation of the tablet within the stomach, causing drug release in the small intestine. The polymeric swelling coating on the surface of tablets similarly causes a delayed release of the contents [91]. Another variation used for delayed release is press coating. An outer layer of dry fine granules is compressed onto the inner tablet core. This method is useful for tablets which contain a core that is unstable with moisture or heat [92]. Pulsatile systems are like delayed release; the principal of pulsatile delivery is delayed onset, lag time, before a burst release of the drug contents. They can also have multiple burst releases with lag times in between. Delivery of the drug is triggered by endogenous signals such as pH change, osmotic pressure, or in other cases, release can be controlled by exogenous signals: ultrasound, magnetic impulse, or electrochemical stimulation [93–96]. A graph depicting drug release over time from various formulations can be seen in Fig. 5. Pulse-in-cap methods utilise a soluble cap which once dissolved causes the plug inside to swell. This plug is then ejected, usually once the capsule reaches the intestines, and leads to a burst release of the drug. These methods of tablet coating and alteration of drug release can be exploited for chronotherapeutic purposes, which will be discussed in further detail.

Delayed release has shown to be beneficial in rheumatoid arthritis, where the modulated release of the glucocorticoid, prednisone, which is used to treat morning stiffness symptoms, was tested in a double-blind, randomised trial. It was found to be more effective at treating symptoms of morning stiffness when given in the modified release form at night versus immediate-release prednisone in the morning [97]. Modified prednisone was given at night for delayed release at 2 am and compared with morning prednisone administration. However, delayed release at night versus regular prednisone treatment at night was not compared; therefore, we cannot conclude that it is the modified delayed release causing an effect or if it is just evening versus morning administration. Nevertheless, this study shows that nighttime dosing of prednisone is more efficacious than morning dosing.

Stroke risk is said to be highest in the morning due to several clinical factors including blood pressure, heart rate, and cerebral blood flow [98, 99]. Therefore, it has been hypothesised that nighttime or evening administration of anti-hypertensives and cardiac therapeutics may be beneficial to counteract the increased incidence of morning stroke. However, there have been no clinical studies to suggest treatment of these morning risk factors will prevent cardiac complications. COER-Verapamil is one of the first delayed-release drug delivery tablets in the USA to be approved by the Food and Drug Administration as a chronotherapeutic for ischemic heart disease and hypertension [100]. COER-Verapamil is an ion channel inhibitor that uses an extended-release system to cause a maximum drug concentration 4–5 h after ingestion. This leads to a high drug concentration in the blood in the early hours of the morning when cardiovascular events are most likely to occur. A large study was carried out longitudinally to compare COER-Verapamil to other cardiovascular drugs, which are usually given as a first line of treatment to patients. This study was terminated early due to sponsor withdrawal; however, after 3 years, it was concluded that COER-Verapamil was not superior to the current recommended first-line cardiovascular treatments [101]. It is essential to note, however, that this study did not include a comparison to treatment at the time of awakening and, as such, cannot be described as a chronotherapy clinical trial. Therefore, more studies are needed to understand COER-Verapamil.

Another variation of delayed delivery is pulsatile delivery systems. Pulsatile systems have been extensively reviewed by Bussemer et al.; we will review some of the main examples in relation to chronotherapy with a focus on immunological diseases [102]. Many pulsatile systems are aimed to have a lag phase long enough to allow the release of the drug within the colon. Usually, drugs have already been absorbed through the gastrointestinal tract before they reach the colon, but with pulsatile drugs, this can be delayed. Modified Pulsincap of ibuprofen and drugs specific for ulcerative colitis, irritable bowel disease, and other colon-specific diseases have been developed [103–107]. Another pulsatile variation which depends on endogenous signals is a hydrogel for type II diabetics, which is responsive to glucose levels in the body and releases insulin when glucose levels are increased [108]. This system is liquid at room temperature and solid with increasing glucose which causes a burst release of insulin. This is an example of a pulsatile system that is responsive to individual biological signals within the body and thus provides a highly personalised approach.

Delayed and pulsatile systems work on very similar principles of lag phase following the release of the drug at the intended time. However, the manufacture of these tablets is lengthier and requires more steps to ensure that drug release will occur at the correct time and the correct dosage.

### Nanomedicine delivery systems

Nanoparticles (NP) are small structures that can be loaded with a drug and offer an alternative form of drug delivery in comparison to tablets as previously discussed. NPs are used due to their small size, increased bioavailability, stabilisation of drugs, and modifiable structure to target them to specific tissues. Drug release from NP relies on several factors, including the polymer, ionic charge, drug concentration and interaction with the polymer itself, the ratio of the compositions, and manufacturing methods [109, 110]. Therefore, there are multiple steps in which modifications can be made or adjusted to tailor nanocarriers towards chronotherapeutic purposes.

Mesoporous and hollow silica NPs are examples of NPs that display a delayed-release profile, or their profile can be modified for a pulsatile release [111–113]. Although NP drug delivery systems such as these have demonstrated controlled delivery and can be targeted to specific tissues, they have yet to be widely explored in the context of chrono-therapeutics [114]. Niosomes are non-ionic surfactant-based vesicles that are structurally similar to liposomes. These vesicles have been investigated as a cancer therapeutic delivery system to deliver at the tumour site with less potent cytotoxic side effects [115]. They may pose as a useful chrono-tailored DDS as they appear to enhance drug entrapment and delay the initial burst release of their contents [116]. Nanocarriers offer great potential in vitro, as a desired release profile is easy to obtain; however, elucidating the exact release profile in vivo can be difficult. The mechanisms of polymeric and lipid NPs have been reviewed by Son et al. on the specific mechanisms of release from various NPs [110]. It has been hypothesised that timed drug delivery of NPs in breast cancer could increase the uptake of the drug into cancer cells and improve the treatment [117]. Timed drug release in this instance is related to monthly timing rather than circadian timing. Vascular endothelial growth factor (VEGF) cycles through the menstrual cycle in women with subsequent increase and decrease in vascular permeability [118]. Timing of drug delivery of cytotoxic chemotherapeutics to coincide with the natural increase of VEGF and therefore, vascular permeability leads to higher drug uptake, particularly within the tumour sites, as they are heavily vascularised. However, further evidence is needed in the literature along with the support of clinical trials to confidently conclude the cycling of VEGF can correspond to enhanced cytotoxicity of the tumour.

### Transdermal delivery

**Fig. 6:**
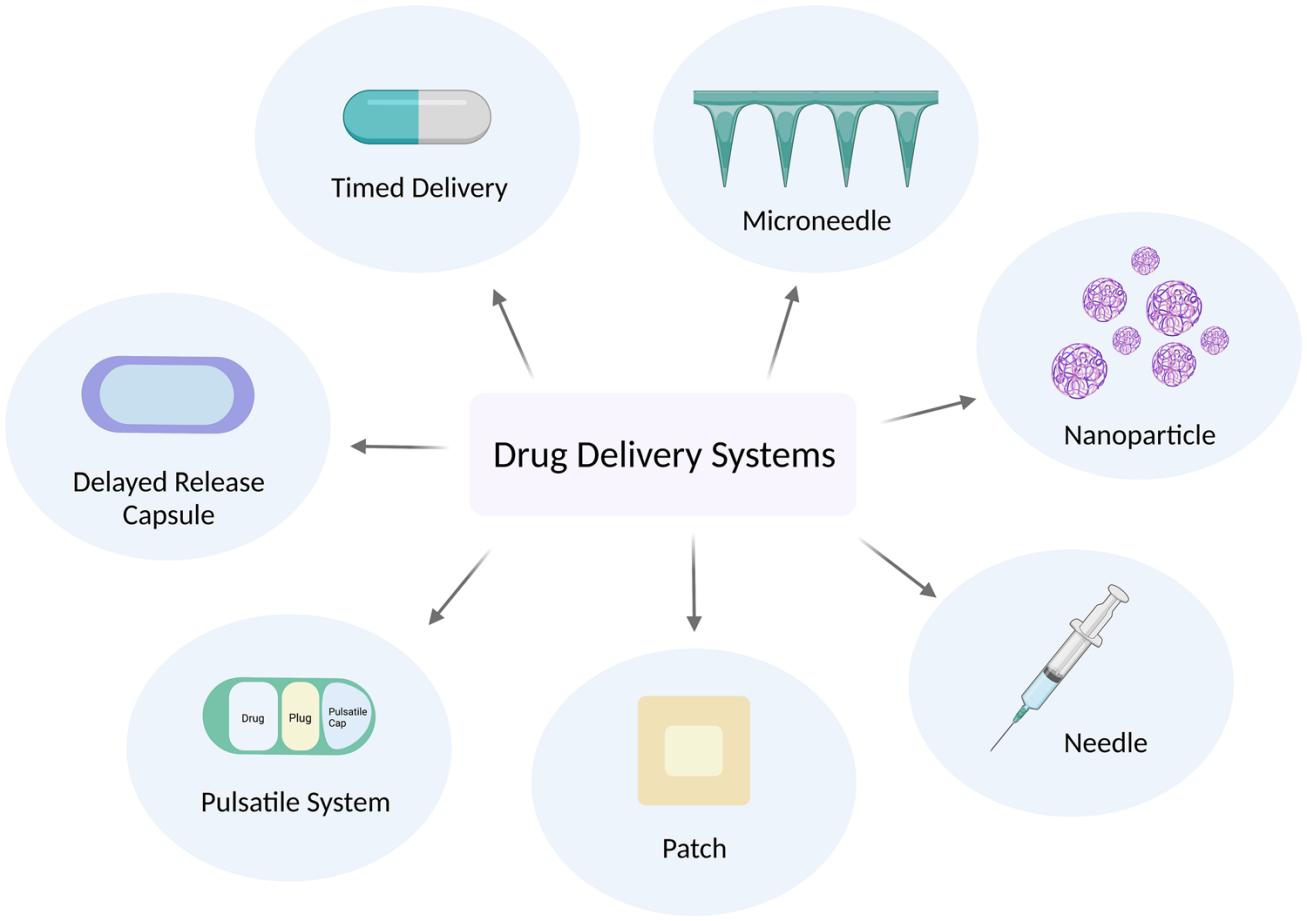
Drug delivery systems. Diagram depicting various drug delivery systems mentioned in this review. Created with BioRender.com

Exploring the skin as a route of administration, particularly for immunological purposes, presents an exciting endeavour as the skin is the largest organ in the body and also has a high proportion of immune cells [119, 120]. The skin has also been shown to be under the control of the clock, with the circadian clock driving rhythms in immune cells, along with cell cycle changes and changes in cell susceptibility to UVB damage [67, 80, 121]. Transdermal delivery offers a unique route of administration than those previously mentioned. Transdermal drug delivery systems include ointments, patches, and microneedles (MN) (Fig. 6). Ointments and patches are more likely to improve patient compliance as they can be easily applied without a medical professional, are painless, and provide an alternative to needles. When applied to the skin, the drug must pass through the stratum corneum into the epidermis and then further into the dermis. Once in the dermal layer, the medication delivered can enter the vasculature or be taken up by resident host immune cells. The stratum corneum acts as a protective layer on the skin and many drugs cannot penetrate through. Therefore, a transdermal delivery system which can breach this layer offers a more practical solution for most drug formulations.

A novel drug delivery system which became popular in the mid-1990s was the MN drug delivery system. Many detailed reviews exist on MN fabrication and current and future applications of MNs [122, 123]. MN patches contain an array of MNs of lengths of 100–1500 µm that pierce the stratum corneum and can deliver drugs through a microchannel. A microchannel is a small channel in the skin that allows compounds that would otherwise be unable to permeate the stratum corneum to enter the skin at different layers ranging from the epidermis to the dermis depending on the length of the microneedle [124]. MNs can be categorised into separate groups: solid, hollow, coated, dissolving, and hydrogel. Different types of MNs offer additional advantages depending on the desired application. Herein, we provide a brief overview of some dissolvable MNs (dMNs) and their potential to be applied with respect to the host’s circadian rhythm.

Dissolvable MNs can offer rapid or slow release depending on the polymer and drug composition, concentration, and ratio. The active ingredient and the polymer must be biodegradable in the dissolving MNs and easily excreted from the body as the entire microneedle is dissolved into the skin. Dissolving MNs, therefore, offer a distinct advantage as they do not produce biohazardous waste. When choosing polymers for dissolvable microneedles, various properties of the polymer and the interactions between the polymer and active ingredient need to be considered. While dissolving MNs require strength to penetrate the skin, they must also readily dissolve. Polyvinyl alcohol (PVA) and polyvinylpyrrolidone (PVP) are two polymers used in dMN applications due to their strength, biodegradability, and overall tolerability in patients. However, a wide range of polymers are being explored for MN applications. Drug loading capabilities of dissolvable MN polymers are an important quality to consider to ensure the correct dosage through intradermal delivery. Recent reviews attached detail the manufacturing protocols of MNs, qualities to consider, and current challenges faced in the field [125–127]. MN and hydrogel MN patches for immunotherapy and chemotherapeutic agents have been described by Wang et al. and Courtenay et al*.* [128, 129]*.* Delivering chemotherapeutic agents trans-dermally with microneedles not only offers more comfort than a needle and syringe method of application but, as previously mentioned, there is a potential benefit to time-of-day administration of chemotherapeutics that could be achieved. There are some clinical trials of dissolving MNs indicating good efficacy in the treatment of keloids and influenza vaccination [130–132]. A clinical trial investigating insulin delivery through dissolving MNs showed faster insulin delivery and higher patient compliance when compared to traditional routes of administration [133, 134]. MNs may also offer a unique function as a bio-responsive agent. Bio-responsive agents act with biological stimuli or abnormalities and therefore could act in accordance with circadian rhythm fluctuations in cytokines, hormones, etc. In terms of microneedles, the release of an active ingredient in line with an increase or decrease in a particular biological marker that is circadian would lead to smart and highly personalised administration of chronotherapeutic drugs. Bio-responsive MNs and polymers that work to release insulin to control glucose levels are a new and interesting area of research [108, 135–137]. The expansion of research into bio-responsive polymers and particles provides an opportunity for the expansion of microneedles as bio-responsive agents, allowing the delivery of drugs at the correct time of day and aiding in chrono-tailoring drug delivery.

Transdermal patches, medicated adhesive patches, can also be used to deliver drugs through the skin. Transdermal patches have been widely used for asthma treatment. As previously mentioned, asthma has been shown to have a strong circadian variation with symptoms being worst in the early hours of the morning. Transdermal delivery of tulobuterol, the beta2-adrenoceptor agonist and bronchodilator, is delivered through a crystal reservoir system. The tulobuterol is in crystal form surrounded by a polymer which controls the release of the drug depending on the polymer properties. The tulobuterol patch has a slow nighttime release to target early morning asthmatic symptoms [138]. The patch is applied at night and provides effective bronchodilation as drug concentration slowly builds over time to be effective during the early hours of the morning when nocturnal symptoms peak. The tulobuterol patch has shown good safety and efficacy in clinical trials, along with greater patient compliance versus inhaled drugs [139]. Thus, this is a good example of the beneficial effects of transdermal delivery to coincide with the circadian rhythm and onset of asthmatic symptoms. The tulobuterol patch is currently on the market in seven countries. This drug delivery system is preferred by patients showing higher patient adherence over inhaled drugs, proving to be an excellent circadian-tailored drug delivery system [140].

### Chrono-vaccination

As discussed, a wide range of immune parameters exist which display circadian rhythms and are affected by circadian gene or environmental perturbation. Thus, the circadian clock plays a significant role in terms of immune cell effector function. At the cellular level, the circadian clock influences immune cell metabolism and morphology. In haematopoietic stem cells, circadian rhythm has been shown to influence migration and release [141, 142]. Within the immune cell populations, macrophage metabolism and immunophenotype have been linked to the circadian clock [18, 68, 143]. Cervantes et al. recently uncovered that DC mitochondrial morphology and metabolism change throughout the 24-h cycle and these changes are dependent on *Bmal1* [66]. This impacts the timing of when to process and present antigens to T cells.

Thus, researchers have begun to explore the impact of the circadian clock within immune cells and its rhythms on the response to vaccination. Herein, we discuss some of the key findings and research which has been carried out to date with respect to circadian timing and vaccination. Time-of-day studies in vaccination have shown differences in response depending on when the influenza or BCG vaccine was administered [144, 145]. A cluster-randomised trial using influenza vaccination compared morning vaccination to that of evening vaccination in older adults. The study demonstrated that morning vaccination induced a higher antibody response in older adults [145]. Enhancing antibody responses in older adults is particularly important as the efficacy of vaccines deteriorates with age [146, 147]. However, the time of sample collection was not noted in this study. As Kurupati et al. recently discussed, the time of sample collection can also influence antibody titres [148]. Similarly, studies assessing the Bacille Calmette-Guerin (BCG) vaccination response demonstrated that the circadian rhythm influenced the trained immune response [144]. De Bree et al. observed this through two studies, a large cohort of 302 individuals and a smaller study of 54 healthy volunteers. In the large cohort, they looked at vaccinations between 8 am and 12 pm, while in the small cohort, vaccination was carried out in the morning and evening. In both cases, peripheral blood mononuclear cells (PBMC) were taken from volunteers before and after vaccination and stimulated with *Staphylococcus aureus* and *tuberculosis*, and cytokine production was measured. Overall, they found that the trained immune response to be higher in PBMCs isolated from the morning cohorts versus the evening cohorts. Further studies have investigated an inactivated SARS-CoV-2 vaccine against Covid-19, with respect to the time of day. Inactivated SARS-CoV-2 vaccination in the morning in health care workers induced higher antibody titres and induced stronger B cell and follicular helper T cells, versus what time of day [149]. An observational study of healthcare workers found significant differences in the anti-spike antibodies against Covid-19 when comparing the Pfizer vaccination versus AstraZeneca at the same times of day [150]. Pfizer’s Covid-19 vaccination is mRNA based, while AstraZeneca is an adenoviral vaccine, thus, highlighting how different vaccination types may be affected by the clock to a greater or lesser extent. Further studies have found that men mount a higher antibody response to the hepatitis A vaccine and influenza vaccine, while females do not have an enhanced antibody response in comparison to men depending on the time of day [151]. Perhaps another cycle which influences the vaccination in women other than the circadian rhythm is the menstrual cycle. There is very little published regarding the effects of the menstrual cycle on vaccination. However, there are studies showing immune fluctuations through the menstrual cycle; therefore, this should also be considered in the effects of circadian rhythm and vaccination in women [152]. Overall, there is evidence which would suggest that the vaccination response is influenced by the circadian clock. Further studies are warranted that take into consideration gender, age, and sample time collection to assess the effects of the circadian clock and what other variables exist which determine the response.

Timing of both drugs and vaccination with respect to circadian rhythm is an important consideration. While further research is warranted to understand the exact time of day of administration of both drugs and vaccination, another key factor to be explored is the best method to administer medications and vaccinations at the correct time of day. If vaccination strategies were to adapt to circadian timing, the traditional route of administration of vaccines would require adaptation. Traditional methods of vaccination through intramuscular injection must be carried out by a healthcare professional, and therefore, it would be difficult to implement vaccination at the correct time of day should that time fall out of hours. Alternative drug delivery systems should be considered for time-of-day vaccination. In clinical trials, CD8 effector cell responses were shown to be increased through intradermal vaccination versus intramuscular route [153]. Fluzone Intradermal Quadrivalent is an intradermal injection influenza vaccination that is currently on the market in the USA for persons aged over 65. Fluzone has shown equivocal or increased immunogenicity when compared to intramuscular vaccination [154–156]. Similar studies and meta-analyses have also shown that intradermal vaccination offers an excellent alternative to the traditional routes of vaccination [157–161]. Clinical trials have also shown self-administration of vaccine patches to be similar to alternative vaccination routes [162–164]. Furthermore, intradermal routes of administration such as MN patches are shown to be preferred by individuals versus an intramuscular route [134, 164]. MNs offer an alternative route of immunisation that is comparable in efficacy to intramuscular which can be self-administered at the correct time of day. Therefore, MN systems may be a useful drug delivery system when considering future strategies to target the circadian rhythm in vaccination.

## Future strategies for chrono-tailored DDS

For transdermal delivery systems such as microneedles, further research around MN manufacture, such as laser ablation, lithography, and micro moulding, need to be adapted for large-scale industrialised production, and good manufacturing practice protocols need to be implemented to manufacture microneedles on a large scale [165–167]. However, as mentioned, MNs are preferred by participants to the intramuscular injection [134, 164, 168]. As MNs are non-invasive and do not cause pain in the same way as conventional needles, they offer an alternative that is preferable [169]. Therefore, they have the potential as chrono-tailored DDS in the future.

While manufactured and used as drug delivery systems, pulsatile systems require more development as chrono-therapeutics. Timed-delivery and delayed-release systems, similarly, offer potential, but further work is needed on the chronobiology of various disease states to implement these drug delivery systems to their highest potential. However, changes to current or new therapeutics for chronotherapeutic delivery will inevitably be met with regulatory hurdles. We refer the reader to the following review which briefly describes some of the regulatory specifications that new chronotherapeutic DDS may face [170].

Chrono-tailored DDS have the potential to enhance drug and vaccination responses. However, many challenges will need to be overcome for this to be translated fully into the clinic. When considering circadian rhythm, each person has a unique chronotype and various genetic and environmental factors which affect their circadian clock and the timing of their circadian rhythm [171]. Currently, there is no simplified approach to accurately determine an individual’s internal circadian rhythm to implement drug delivery at their exact optimal time of day. Wittenbrink et al. describe a single blood sample which can determine the internal circadian time [172]. While being able to determine an individual’s internal circadian time would be beneficial to chrono-tailored DDS, the use of the described blood test would pose its own barriers, including economic and logistic implementation. We need to further understand drug targeting across the 24-h cycle in healthy individuals to directly compare to our disease cohorts. A wider knowledge of drug targets and pharmacokinetics and dynamics concerning the clock is essential. Our basic knowledge of the 24-h in relation to healthy individuals is key. Further, a deeper understanding of the chronobiology of various disease states will allow us to implement many of these chrono-tailored DDS, to maximise patient benefits and minimise risk.

Patient benefit is at the forefront of translational research. For future studies, it will be essential that the timing of drug delivery and sample collection is considered. It is important to keep in mind that the administration of therapeutics at the incorrect time may result in toxic side effects. New therapeutics must not be simply disregarded due to adverse events if administration at the correct time-of-day could reduce these effects to acceptable levels. A simplified solution in modifying administration times and varying the delivery system has the potential to lead to more effective therapeutics and their approval. Optimising existing medications with future circadian research may lead to enhanced drug benefits and patient compliance. At a minimum for clinical trials, the time of day of drug administration or any intervention should be recorded, along with the time of sample collection. Sleep-wake cycle and timing of nutrition habits are also important to take into consideration. Information regarding the aforementioned is vital to determine the impact of circadian rhythm on drug delivery in trial and to allow the field of chrono-tailored drug delivery systems to advance.

## Conclusions

Chrono-tailored DDS show great potential in enhancing the efficacy of therapeutics and drug delivery with the possibility of minimising patient side effects. Recent advances in the field have led to the development of innovative drug delivery systems that can release drugs in a controlled and timely manner, according to the body’s circadian rhythm. As discussed, these systems have shown potential in treating cancer, immunological diseases, and neurological disorders.

The future of the chrono-tailored DDS field holds promise, with continued research in the development of new technologies and strategies to optimise drug delivery on a circadian basis. Personalised medicine to tailor drug delivery to a patient’s circadian rhythm, along with incorporating new nanocarriers to develop more efficient drug delivery systems, have the potential to advance the field even further. As mentioned, chrono-tailored DDS is incredibly interdisciplinary, and consequently, the progression of chrono-tailored DDS relies on the continued development and collaboration of many different areas of scientific research to advance. Despite the significant progress in recent years, there are still many challenges. An important aspect that needs to be addressed is a clearer understanding of the mechanisms underlying the circadian rhythm, particularly in disease pathologies. Similarly, developing more cost-effective drug delivery systems that can be tailored easily to the circadian rhythm is needed.

Overall, the field of chrono-tailored DDS represents a rapidly evolving and exciting area of scientific research. It holds great potential for improving therapeutic responses and patient outcomes. With continued research and innovation over the coming years, we can expect significant advances in the development of new and novel chrono-tailored DDS.

## Data Availability

N/A

## Declarations

### Data collection methods

The authors used databases such as PubMed with keywords such as circadian rhythm and drug delivery to define the current literature on the subject. Once an understanding of the current literature was defined, further research into individual topics defined within this review was carried out, including delayed release, transdermal delivery, vaccination etc. Journals, books, and other sources were also used to find academic articles related to the topics of circadian rhythm and drug delivery.
